# Effect of Traverse Speed Variation on Microstructural Properties and Corrosion Behavior of Friction Stir Welded WE43 Mg Alloy Joints

**DOI:** 10.3390/ma16144902

**Published:** 2023-07-09

**Authors:** Yusra Saman Khan, Mustufa Haider Abidi, Waqar Malik, Nadeem Fayaz Lone, Mohamed K. Aboudaif, Muneer Khan Mohammed

**Affiliations:** 1 Department of Mechanical Engineering, Jamia Millia Islamia, New Delhi 110025, India; 2 Raytheon Chair for Systems Engineering, Advanced Manufacturing Institute, King Saud University, Riyadh 11421, Saudi Arabia

**Keywords:** Mg WE43, friction stir welding, polarization curves, corrosion rate, rare earth elements, precipitates

## Abstract

The growing demand for Magnesium in the automotive and aviation industries has enticed the need to improve its corrosive properties. In this study, the WE43 magnesium alloys were friction stir welded (FSW) by varying the traverse speed. FSW eliminates defects such as liquefication cracking, expulsion, and voids in joints encountered frequently in fusion welding of magnesium alloys. The microstructural properties were scrutinized using light microscopy (LM) and scanning electron microscopy (SEM). Additionally, the elemental makeup of precipitates was studied using electron dispersive X-ray spectroscopy (EDS). The electrochemical behavior of specimens was evaluated by employing potentiodynamic polarization tests and was correlated with the microstructural properties. A defect-free weldment was obtained at a traverse and rotational speed of 100 mm/min and 710 rpm, respectively. All weldments significantly improved corrosion resistance compared to the base alloy. Moreover, a highly refined microstructure with redistribution/dissolution of precipitates was obtained. The grain size was reduced from 256 µm to around 13 µm. The corrosion resistance of the welded sample was enhanced by 22 times as compared to the base alloy. Hence, the reduction in grain size and the dissolution/distribution of secondary-phase particles within the Mg matrix are the primary factors for the enhancement of anti-corrosion properties.

## 1. Introduction

The need for weight reduction in the aviation industry has grown manifold over recent years, driven by stringent CO_2_ emission regulations and the need to increase fuel efficiency [1]. Due to its low specific weight and high strength-to-weight ratio, Magnesium (Mg) is a potential alternative to Al alloys for aeronautical components [2]. The density of Mg is 77% and 33% lower compared to steel and aluminium, respectively [3]. Over the last decade, magnesium alloys have been used to account for more than 15% of the weight of vehicles [4]. Some of the applications in the automobile sector include clutches, crankcases, valve covers, and cylinder heads. Other applications include steering wheels, seat frames, and alloy wheel rims. Furthermore, these materials also find applications in defense and armor due to their shielding ability and noise-damping capacity [2,5]. Mg alloys containing rare-earth (RE) elements have gained prominence because of their superior properties, such as creep and corrosion resistance, ductility, and strength compared to other magnesium alloys [2,6]. RE elements (Mg-4Y-3X-Zr, X = Gd/Nd) tend to form a safeguarding layer over the surface, thereby inhibiting the corrosion rate [7,8]. Among RE-based Mg alloys, the ASTM designated WE43 alloy is regarded as a high-strength alloy that can be employed at as high as 250 °C. These characteristics make it most sought after for several high-end applications, such as transmission and power systems of military aircraft and helicopters. Ever since Mg has been categorized as inflammable-safe, its adoption in several sectors has increased but it mainly remained focused on AZ and AM series alloys. However, the WE43 series is superior to the AZ31, AM60, AZ91, and AM50 alloy series in terms of corrosion resistance. Its biocompatibility also makes it popular in other exotic applications, such as bio-absorbable implants, including screws, reinforcement/support plates, and stents [9,10,11]. The expanse of application domains is directly coupled with the welding of alloys. A critical review of the literature carried [2,12,13] out as part of this work reveals that despite far better properties of WE43 compared to common Mg alloys of the AM/AZ series, WE43 has not been extensively used.

Despite the improved anti-corrosion properties of the WE43 Mg alloy, it is still susceptible to corrosion due to precipitates, secondary phase particles (SPPs), and grain size. The main concern is the higher gain boundary energy and galvanic cell formation around the precipitates and SPPs. Several strategies have been used to improve Magnesium’s corrosion resistance. The use of protective coatings is an effective method against the external environment [10]. Alloying with high-purity elements has also been used to enhance corrosion resistance [14]. Other strategies deployed to enhance corrosion resistance include surface modification by ion implementation, laser annealing, and grain refinement [15].

The growth of Mg alloys in aviation, automobiles, and other key sectors necessitates its joining with self and other interfaced materials in comparable volume to its use. Fusion welding of Mg alloys frequently results in expulsion, voids in weldment, and liquefication cracking [16,17]. Friction stir welding (FSW), a solid-state process, eliminates the aforementioned solidification-related problems; it is energy efficient and regarded as a green welding technique [18,19]. FSW is a promising welding technique to join similar as well as dissimilar materials [20,21,22,23]. FSW also refines and modifies the grain structure, often leading to the redistribution and dissolution of SPPs [24]. The grain morphology modification improves several mechanical properties, including tensile strength, hardness, ductility, and corrosion resistance [25]. The stirring action under frictional heat is known to modify basal texture and may result in the dissolution or redistribution of SPPs depending on the prevailing temperature. The stirring action during FSW and friction stir processing (FSP) refines and redistributes the SPPs and helps improve corrosion resistance [26,27,28]. FSP has been used to modify the microstructure of FSW materials. For instance, Mehdi and Mishra [29] successfully performed the FSP on dissimilar aluminium joints welded by Tungsten inert gas welding and reported that, at higher tool rotational speed, tensile strength and hardness can be increased. Charandabi et al. [30] also utilized the FSP to modify the mechanical and microstructural properties of an Al-Si alloy. The results showed enhancement in mechanical properties and the microstructure was modified by eliminating the porosity, dendritic structure, and segregation of particles. Moreover, the SPPs were redistributed uniformly. The effect of refinement and microstructural morphology on corrosion behavior is presently attracting colossal interest [31].

Some works related to the corrosion behavior of the WE43 series have been reported. For instance, Pereira et al. [8] studied the corrosion rate of Mg-WE43 and pure Mg alloy by immersing the specimens in 0.6 M brine solution. They observed that the corrosion rate of pure Magnesium was much higher compared to WE43. The enhanced corrosion resistance in WE43 was attributed to the development of a protective layer over the surface. Additionally, in their study, Chu and Marquis [32] investigated how the heat-treated microstructure of WE43 affects the corrosion rate and found that age hardening can lead to increased corrosion resistance. Yang et al. [33] also compared the corrosion rate of WE43 alloy in both as-cast and heat-treated conditions and demonstrated that the eutectic phase in the as-cast structure damages the protective layer, thereby accelerating the corrosion rate. The property evolution, apart from process parameters, is also greatly affected by the thickness of the cross-section being welded. The thickness adds to the microstructural and thermal profile heterogeneity, significantly affecting the properties of and around the joint [34]. Whereas high-end industrial applications, e.g., space/aerospace and aircraft, call for thick sections, the reported literature is mainly focused on relatively thin sections [35]. The microstructural, corrosion property evolution, and their correlation for thick section welding prove to be more useful but, unfortunately, very acutely reported. To the best knowledge of the authors, the effect of grain size modification and redistribution of SPPs attained by FSW on the corrosion rate is not yet reported in the literature. Importantly, while Mg alloys are marked for poor corrosion resistance, the WE series Mg alloys are the recent class of materials which are developed for improved corrosion behavior. The welding modifies the corrosion behavior and, in the case of welding of the thick WE series, which is acutely reported, the investigation on corrosion behavior is valuable in better understanding the weld characteristics of this recent class of material. This maiden work investigates the effect of traverse speed in a through-thickness FSW WE43 Mg alloy with 11.5 mm thick plates and simultaneously evaluates the effect of microstructure evolution on the corrosion rate of the welded specimens and the base alloy using potentiodynamic polarization tests.

## 2. Materials and Methods

**Figure 1:**
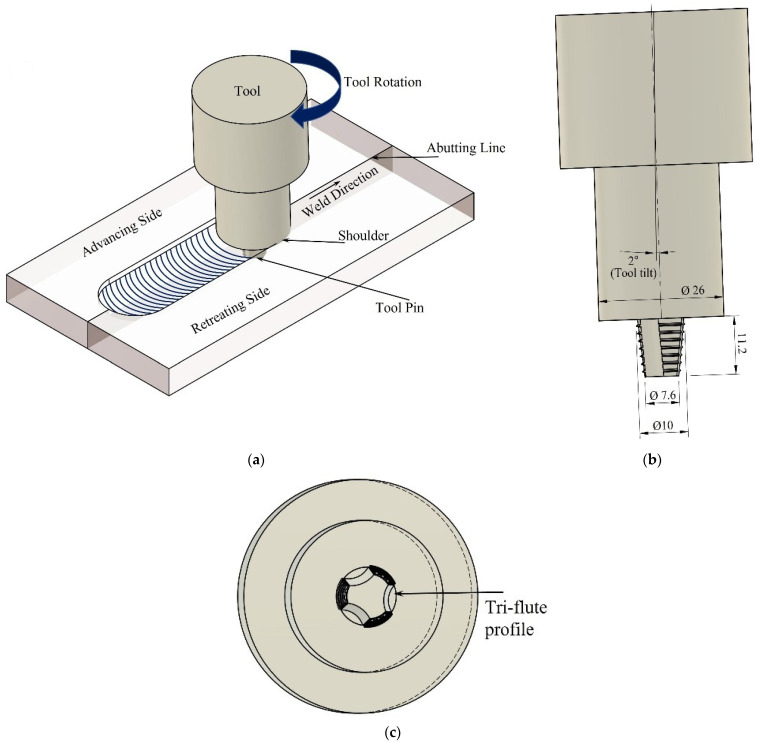
(**a**) FSW process schematic, (**b**) tool profile used, and (**c**) bottom view of tapered cylindrical cam tri-flute tool probe.

The as-cast Mg-WE43 alloy plates, having a thickness of 11.5 mm, were butt welded by carrying out FSW. The FSW setup, experimental tool design, and bottom view of the tool probe are shown in Figure 1. Before welding, the plates were cleansed with acetone. The FSW was carried out on a retrofitted vertical milling machine with a high-speed steel (HSS) tool having a tapered cam tri-flute profile. The plates were tightly clamped in a work fixture for easy processing or joining of the plates. The tool dimensions were kept constant with a 26 mm shoulder diameter, 11.2 mm pin length, 10 mm pin root diameter, and 7.6 mm pin tip diameter.

The process parameters employed to carry out the experimentation are enlisted in Table 1. Table 2 shows the elemental makeup of the Mg base alloy.

For metallographic examination, the samples of the desired shape and size were wire drawn via a wire electric discharge machining (WEDM). After extraction, the samples were polished with SiC abrasive papers (procured from Akshara polishing, Faridabad, India) with grit sizes ranging from 100 to 2500, followed by polishing on a diamond velvet cloth as per the standard metallographic practices. After polishing, the samples were etched with 2.5 mL acetic acid, 3 gm picric acid, 50 mL ethanol, and 5 mL water for 30 s, and then properly rinsed with demineralized water and dried (chemicals were obtained from Insta Chemi, Noida, India). Scanning electron microscopy (SEM) using secondary electrons (SE) with an acceleration potential of 10 kV, spot size of 50 nm, and vacuum pressure of 1.81 × 10^−5^ mbar and light microscopy (LM) (provided by HD services technology, Haryana, India) were used to scrutinize the grain morphology of the base alloy and the welded sample. The elemental makeup of secondary phase particles (SPPs) was examined using energy dispersive X-ray spectroscopy (EDS) (Carl Zeiss Microscopy GmbH, Oberkochen, Germany). The line-intercept method was employed to measure the grain size using a metallurgical image analysis software (MIAS), namely ImageJ (version-2).

Furthermore, the polarization tests were performed on both the base metal (BM) and the welded specimens using a three-electrode configuration (cell), which was connected to the Zive SP1 Potentiostat (obtained from TechScience Services, Guindy Chennai, India). The samples were taken as the working electrode and were wire-drawn from the transverse section in the welding direction. After extraction, the samples were polished with abrasive papers and diamond velvet cloth, followed by ultrasonic cleansing with ethanol before being subjected to electrochemical testing. In addition, a circular area of 1 cm^2^ was subjected to chemical testing. Saturated calomel and platinum electrodes were also used for reference and counter electrode purposes, respectively. The corrosion rate was evaluated by an electrochemical technique called Tafel extrapolation in which the polarization plot curves were obtained at a scan rate of 0.16 mV/s between potentials ranging from −400 mV to +400 mV with the incubation period of 2 h (7200 s) in 3.5 %wt. brine solution as an electrolyte at 25 °C (prepared in 300 mL of demineralized water to stabilize the initial conditions). Using the Tafel extrapolation technique, the corrosion current density (I_corr_) and the corrosion potential (E_corr_) were calculated. Lastly, the samples after corrosion were analyzed using light micrographs.

## 3. Results and Discussion

This section describes the experimental results, their elucidation, as well as the experimental inferences that can be drawn.

### 3.1. Grain Morphology Analysis Using Macro and Micrographs

**Figure 2:**
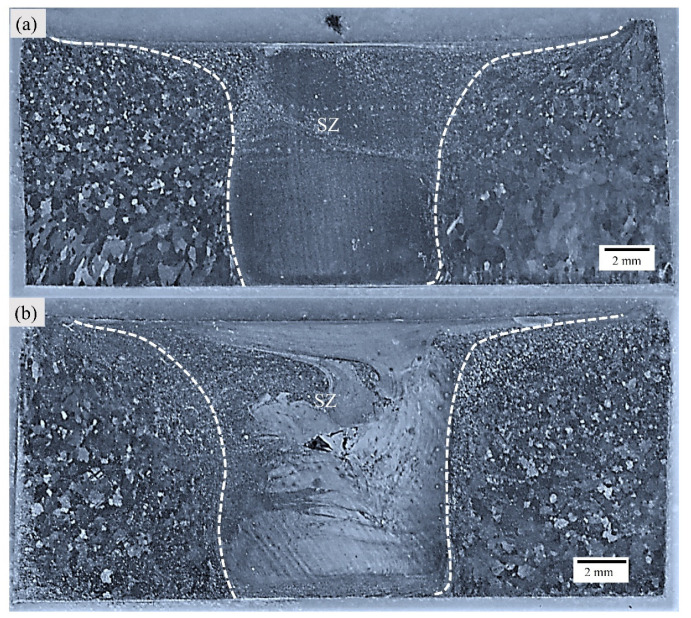
Macrograph for Exp. 1 (**a**) and 2 (**b**) [39].

The FSW leads to the formation of distinct microstructural zones possessing unique features throughout the processed region due to varying strain rates and heat input during welding. Figure 2a,b capture the transverse section’s optical macrographs, corresponding to Exp. 1 and Exp. 2, respectively. The characterized zones are well-demarcated. The stir zone (SZ) is subjected to severe plastic deformation (SPD) and is in close proximity to the heat source, thereby resulting in dynamic recrystallization (DRX) of grains in this region [36]. The SZ is encapsulated by a thermo-mechanically affected zone (TMAZ); this region undergoes partial plastic deformation and lower thermal cycles compared to SZ [37]. The grains are usually elongated in shape. TMAZ is followed by a heat-affected zone (HAZ), which is characterized by coarse grain morphology as this zone is subjected to cold thermal cycles only [38].

The macrograph corresponding to Exp. No. 1 is free of any defect; however, the macrograph of Exp. No. 2 shows a tunneling defect [39]. The existing literature suggests that a tunneling defect is usually formed at the bottom of the weld’s advancing side (AS). However, in the present case, the defect is formed at the transition of the pin-affected stir zone (PASZ) and shoulder-affected stir zone (SASZ). These defects are sensitive to heat input and are directly controlled by the welding parameters. In the case of thick plates, the heat gradient is difficult to maintain across the weld depth. In the case of Exp. No. 2, the weldment is subjected to high heat input as compared to Exp. No. 1. The higher value of the pseudo heat index (w^2^/v) indicates higher heat input. Due to difficulty in maintaining flow synergy across the hotter SASZ and colder PASZ, the defect is formed at this location [39].

**Figure 3:**
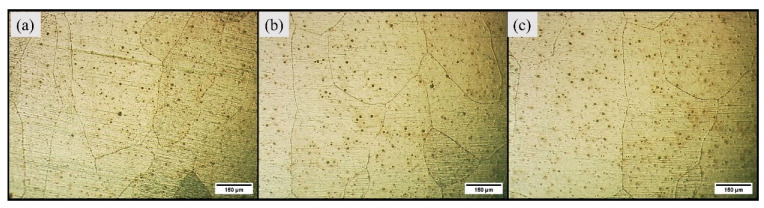
Light micrographs of base alloy (**a**) Region-1, (**b**) Region-2, and (**c**) Region-3.

**Figure 4:**
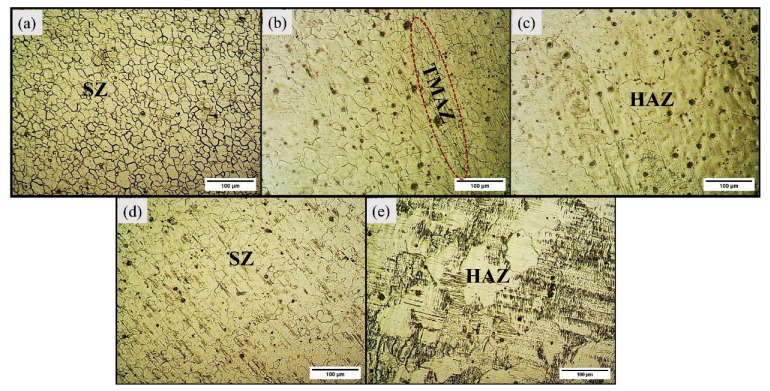
Light micrographs of weld sample 1 (**a**–**c**) and weld sample 2 (**d**,**e**).

The light micrographs for base alloy are captured in Figure 3. The base alloy has a coarse grain structure, and the average grain size measured was 256 µm. The visual inspection of light micrographs for Exp. 1 and Exp. 2 show extensive grain refinement in all the characteristic zones (refer to Figure 4). The average grain size measured in SZ of Exp. No. 1 and Exp. No. 2 are 12.037 µm and 14 µm, respectively.

The grain size in SZ is around 21 and 18 times lower in Exp. 1 and 2, respectively, as compared to the as-casted base alloy. Also, the visual inspection of TMAZ and HAZ shows a refined grain structure. The ultra-refinement in SZ is due to the SPD-assisted DRX, while the TMAZ experienced refinement occurs due to the induced strain rates [40]. The differences in the grain size of SZ in Exp. 1 and 2 are due to the heat input, which is attributed to the variation in traverse speed involved. The grain size has increased with a decrease in traverse speed and is in agreement with the existing literature [41]. When the traverse speed increases from 80 mm/min to 100 mm/min, the frictional heat reduces, consequently decreasing the average distance between the two adjacent particles [42]. At lower traverse speed, the stirring mm/revolution increases, resulting in more heat generation, thereby increasing the grain size. In metals, the corrosion resistance is directly influenced by the grain size. The higher the grain size, the more the corrosion rate [43].

### 3.2. Chemistry and Distribution of Secondary-Phase Particles Using SEM–EDS

**Figure 5:**
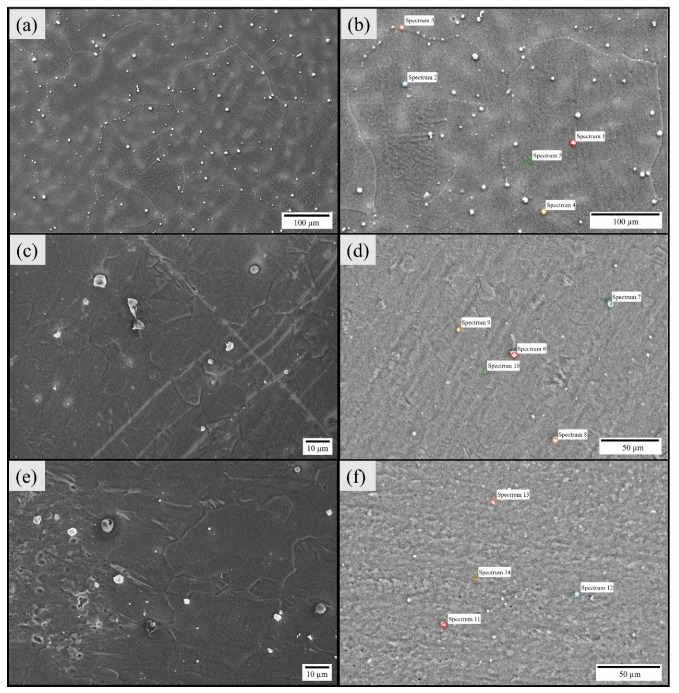
SE micrographs with EDS point spectrum marked: as-cast base alloy (**a**,**b**), Exp. No. 1 (**c**,**d**), and Exp. No. 2 (**e**,**f**).

The secondary electron (SE) micrographs of the as-cast base alloy and the stir zone of specimens corresponding to weld sample I and weld sample II are shown in Figure 5. The micrographs of base alloy captured by Figure 5a,b reveal large grains and the presence of β secondary phase particles (SPPs) both within and at the grain boundaries and rectangular plate-like intermetallic compounds (IMCs). Furthermore, the Mg-containing Nd results in the precipitation of IMCs within the grains and at the grain boundaries of the α-Mg matrix during casting [44]. Pure REs also segregate during the solidification process. Moreover, the size of SPPs is large as compared to welded samples. The energy dispersive X-ray spectroscopy (EDS) point spectrum results of these particles are shown in Table 3. The EDS point spectrum pertaining to 1, 2, 3, 4, and 6 were directly taken on the phases found within the matrix, thereby showing discrepancy in the chemical composition with the rest. The EDS results confirm the elemental makeup of the SPPs. The distribution of these particles is more pronounced on the grain boundaries. In addition, the micrographs of SZ of Exp. 1 and 2 are represented by Figure 5c,d and Figure 5e,f, respectively. The SZ has ultra-refined grains, and the SPPs are accumulated only at grain boundaries (refer to Figure 5c,e). The stirring action during welding results in fragmentation and redistribution of SPPs. These fragments’ phases are smaller in size and have a tendency to accumulate at the grain boundaries upon plasticization [1].

The presence of precipitates greatly affects the corrosion rate and is generally responsible for the development of a galvanic couple, which consequently increases the corrosion rate [1]. The as-casted base alloy has a high density of precipitates and the distribution is mainly non-homogenous. The welded specimens undergo high plastic deformation coupled with high heat input, thereby resulting in the dissolution of SPPs. However, the accumulation of precipitates is also observed at the grain boundaries. Overall, the RE elements (Y/Gd) have a strong affinity to accumulate at the grain boundaries upon plasticization [45].

### 3.3. Electrochemical Analysis Using Potentiodynamic Polarization Measurements

In the corrosion test, the corrosion potential (E_corr_) and corrosion density (I_corr_) were estimated by the potentiodynamic polarization test curves in which the cathodic and anodic branches were obtained. The sample surfaces were exposed to a NaCl solution of 3.5 %wt. for 7200 s to obtain the open circuit potential (OCP), and the potentiodynamic measurements were carried out as per standard practices. The I_corr_ and E_corr_ were attained by the intersection of the extrapolated curves of the anodic and cathodic potentiodynamic polarization branches [46,47].

**Figure 6:**
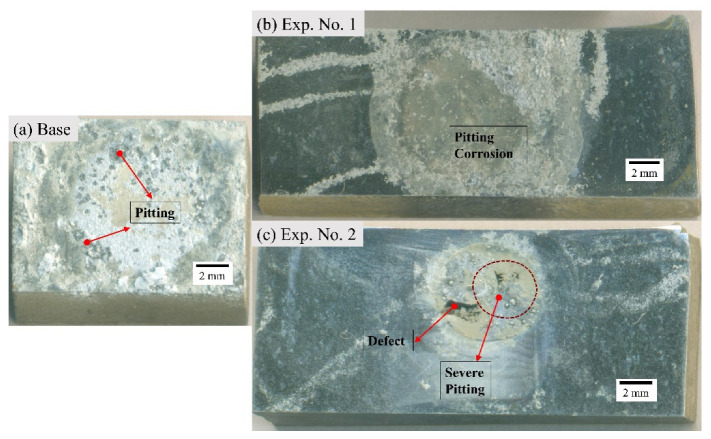
The macrographs for (**a**) base alloy, (**b**) Exp. No. 1 (weld sample I), and (**c**) Exp. No. 2 (weld sample II).

**Figure 7:**
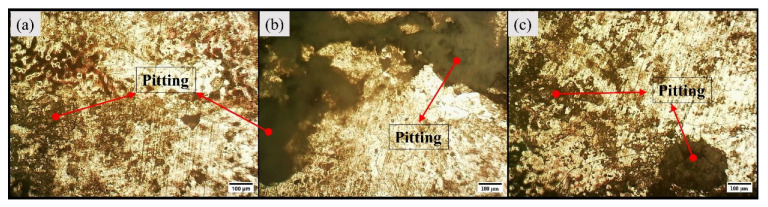
Optical micrographs of base alloy after corrosion (**a**) base alloy, (**b**) Exp. No. 1 (weld sample I), and (**c**) Exp. No. 2 (weld sample II).

**Figure 8:**
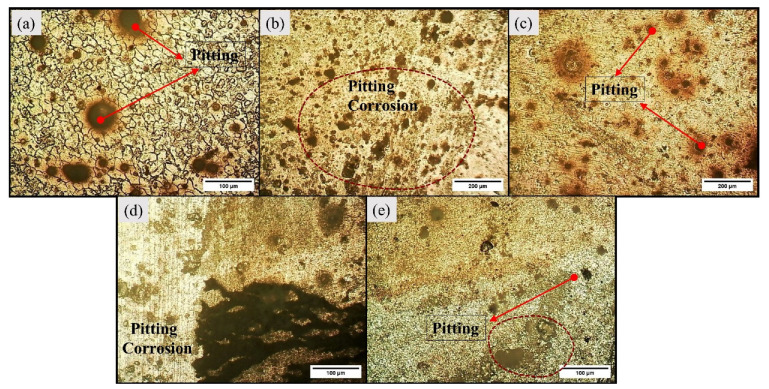
Microstructure of weld sample 1 (**a**–**c**) and weld sample 2 (**d**,**e**) after corrosion.

The macrographs of the transverse section of the BM and the welded samples after corrosion are depicted in Figure 6. All the specimens have corroded; however, the base alloy has undergone severe pitting in comparison to samples corresponding to Exp. No. 1 and 2. The micrographs of base metal and samples from Exp. No. 1 and 2 are represented by Figure 7 and Figure 8, respectively. From the visual inspection of these samples, it is evident that the corrosion in the base alloy is more prominent. Moreover, there is less observable corrosion in the sample corresponding to Exp. No. 1 compared to the sample corresponding to Exp. No. 2.

**Figure 9:**
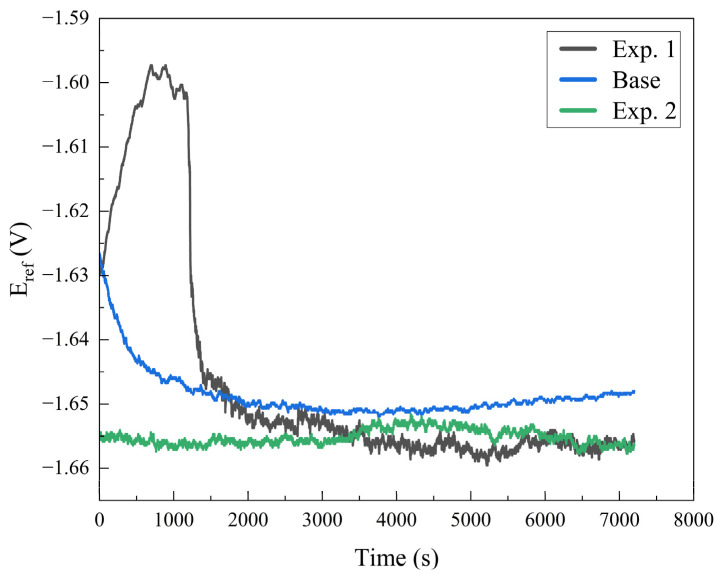
OCP curves for base alloy, weld sample I and II.

The open circuit potential (OCP) curves for base alloy and welded samples are shown in Figure 9. For the base alloy, the OCP dropped in the first 1000 s, which indicates an increase in the anodic reaction and, therefore, suggesting more dissolution of the base alloy. After 1000 s, the anodic and cathodic reaction rates became almost equal (reached equilibrium) and may have formed a passivation layer afterwards. In the case of weld sample I, for the first 1000 s, a decrease in the anodic reaction and an increase in the cathodic reaction was observed, indicating the formation of passivation layer and the evolution of sufficient oxygen. The weld sample II behaved ideally. For the initial seconds of the test, no shift in potential was observed, meaning that the anodic and cathodic reactions have the same rate.

**Figure 10:**
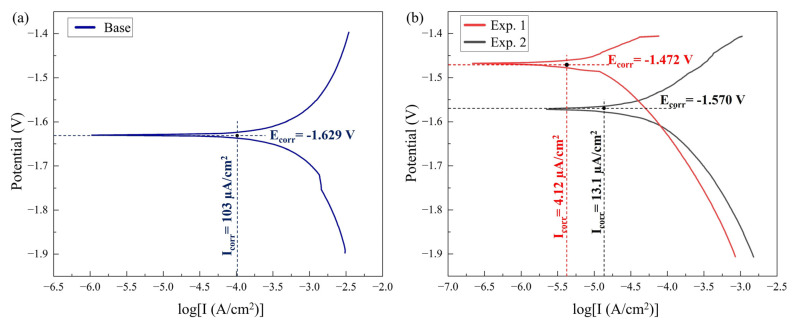
Potentiodynamic polarization curve for (**a**) base alloy and (**b**) weld samples I and II exposed to 3.5 %wt. electrolyte.

Figure 10 illustrates the potentiodynamic curves obtained for the base metal and welded samples from Exp. No. 1 and 2 (samples I and II, respectively). The I_corr_, E_corr_, and corrosion rate (in mils penetration per year) of the BM and welded samples (I and II) are given in Table 4. According to the existing corrosion thermodynamics research, the corrosion potential expresses the degree of difficulty of corrosion. It implies that with a higher positive (or less negative) corrosion potential, the material is more resistant to corrosion and, according to corrosion kinetics, the higher the corrosion density, the lower the exhibited corrosion resistance [48]. The welded samples (I and II) showed lower corrosion current density and higher corrosion potential compared to the base metal (refer to Table 4). The corrosion rate obtained for the base metal is 95.604 mpy, while for the welded samples I and II, it is 4.315 mpy and 13.69 mpy, respectively. Hence, the potentiodynamic results show that the welded samples are more resistant to corrosion.

The corrosion rate of the metal Is controlled by the redox reaction, with a higher anodic reaction rate representing faster oxidation of Mg alloy by promoting the yield of Mg^2+^ ions, thereby causing the dissolution of the alloy at a higher rate, consequently depicting the lower corrosion resistance and vice versa. On the other hand, the cathodic reaction controls the yield of oxide/hydroxide ions. The higher the reaction rate, the more likely the formation of oxide/hydroxide ions takes place. Thus, Mg^2+^ ions react with oxide/hydroxide ions and form a passive protective layer of MgO/Mg(OH)₂, which prevents further corrosion. The redox reactions are given as follows:

$$\mathrm{Anodic}\;\mathrm{reaction}:\;\mathrm{Mg}\to {\mathrm{Mg}}^{2+}+{2e}^{-}$$

$$\mathrm{Cathodic}\;\mathrm{reaction}:\;2H_{2}O+{2e}^{-}\to 2{OH}^{-}+H_{2}$$

$$\mathrm{Overall}\;\mathrm{reaction}:\;\mathrm{Mg}+{2H}_{2}O\to {\mathrm{Mg}(\mathrm{OH})}_{2}+H_{2}$$

here, the anodic and cathodic reaction rates are determined by the slopes of the anodic and cathodic curves in the Tafel plots (refer to Figure 10): the higher the slope of the corresponding curve, the higher the reaction rate and vice versa.

There are two possible reasons for this type of corrosion behavior; first, the grain size and, second, the distribution/dissolution of secondary-phase particles (SPPs) within the substrate. The base metal has large grains, and there is a larger presence of solute precipitates compared to welded samples. This large grain size does not allow the development of a protective layer over the surface [6]. However, a reduction in grain size results in more grain boundaries, which inhibits the formation of a galvanic couple [31,43]. Moreover, the presence of SPPs accelerates the corrosion rate by forming the galvanic couple with the Mg matrix, possessing higher corrosion potential than the matrix, which leads to more localized corrosion in the form of pitting corrosion, as observed in the base alloy [1]. After FSW, SPPs are redistributed and fragmented; hence, the corrosion rate decreases or the preferred sites of grain boundaries for pitting corrosion decrease [49]. The localized sites of corrosion, which are at the grain boundaries decreased and, thereby, their detrimental effect has been significantly reduced. The intensity of the formation of the galvanic couple has diminished. The higher corrosion rate in weld sample II as compared to weld sample I is preferably due to the difference in grain size and due to the presence of the defect.

## 4. Conclusions

This experimental work evaluates the electrochemical/corrosion behavior of friction stir welded WE43 Mg plates using potentiodynamic tests and simultaneously evaluates the impact of welding speed on the corrosion rate. The microstructural properties were scrutinized using light microscopy and scanning electron microscopy and were co-related with the corrosion behavior of the specimens. The following outcomes are drawn based on the results obtained:

1. A defect-free weldment was obtained at a traverse and rotational speed of 100 mm/min and 710 rpm, respectively. With a decrease in the traverse speed, a tunneling defect was obtained at the transition of the pin-affected and shoulder-affected stir zone.
2. Ultra-refined grains were observed in both weldments. The average grain size decreased from 256 µm (base metal) to 12.037 µm and 14 µm in weld I and weld II, respectively. As traverse speed decreased, the grain size of the welded samples increased. The grains were more refined and equiaxed at a higher traverse speed compared to a lower traverse speed.
3. The base alloy had a significant presence of secondary-phase particles. These precipitates were redistributed within the Mg substrate after performing the FSW.
4. A substantial enhancement in corrosion resistance was obtained in both weldments in comparison to the base alloy. The corrosion rate for the defect-free welded sample was 22 times lower as compared to the base alloy.
5. The primary reasons for the enhancement in corrosion resistance are the decrease in grain size and the dissolution of solute particles within the substrate. A larger grain size is more prone to corrosion. Furthermore, the presence of precipitates/solute particles intensifies the formation of the galvanic couple, thereby increasing the corrosion potential.
6. Corrosion resistance decreases with an increase in grain size in the case of the welded specimens.

## Figures and Tables

**Table 1:** Process parameters utilized for FSW joints of WE43 Mg alloy.

Experiment No.	Weld Sample	Rotational Speed (rpm)	Traverse Speed (mm/min)	Tilt Angle
1	I	710	100	2⁰
2	II	710	80	2⁰

**Table 2:** Major Elemental composition (in %wt.) of WE43 alloy.

Elements	Mg	Y	Gd
%wt.	93	4	3

**Table 3:** EDS point spectrum elemental makeup.

Element (%wt.)	1	2	3	4	5	6	7	8	9	10	11	12	13	14
Mg	85.5	76.1	80	81.1	96.5	46.1	91.9	92.5	79.8	92.7	92.3	92.6	92.3	92.4
Y	6.8	14.4	9.7	9.7	1.7	43.5	4.2	3.5	16.6	3.0	3.8	3.9	3.7	3.2
Zr	1.2	1.4	1.1	1.4	0.3	1.0	0.7	0.5	1.0	0.3	1.4	0.5	0.5	0.5
Gd	6.5	8.2	9.1	7.8	1.5	9.4	3.2	3.4	2.7	3.9	3.4	3.0	3.5	3.9

**Table 4:** Electrochemical parameters of the tested samples exposed to 3.5% wt electrolyte.

Specimen	E_corr_ (V)	I_corr_ (µA/cm^2^)	CR (mpy)	CR (mmpy)
Base alloy	−1.629	103	95.604	2.428
Weld I	−1.472	4.12	4.314	0.109
Weld II	−1.570	13.1	13.68	0.034

## Data Availability

All the relevant data are available in the manuscript.
